# Liquid–Liquid Phase Separation of the Intrinsically Disordered Domain of the Fused in Sarcoma Protein Results in Substantial Slowing of Hydration Dynamics

**DOI:** 10.1021/acs.jpclett.3c02790

**Published:** 2023-12-06

**Authors:** Carola S. Krevert, Daniel Chavez, Sayantan Chatterjee, Lukas S. Stelzl, Sabine Pütz, Steven J. Roeters, Joseph F. Rudzinski, Nicolas L. Fawzi, Martin Girard, Sapun H. Parekh, Johannes Hunger

**Affiliations:** a Department of Molecular Spectroscopy, Max Planck Institute for Polymer Research, Ackermannweg 10, 55128 Mainz, Germany; b Department of Polymer Theory, Max Planck Institute for Polymer Research, Ackermannweg 10, 55128 Mainz, Germany; c Department of Biomedical Engineering, The University of Texas at Austin, 107 West Dean Keeton Street, Stop C0800, Austin, Texas 78712, United States; d KOMET 1, Institute of Physics, Johannes Gutenberg University, Staudingerweg 7, 55099 Mainz, Germany; e Faculty of Biology, Johannes Gutenberg University Mainz, Gresemundweg 2, 55128 Mainz, Germany; f Institute of Molecular Biology (IMB), Ackermannweg 2, 55128 Mainz, Germany; g Department of Chemistry, Aarhus University, Langelandsgade 140, 8000 Aarhus C, Denmark; h Department of Anatomy and Neurosciences, Amsterdam UMC, Vrije Universiteit, De Boelelaan 1108, 1081 HZ Amsterdam, The Netherlands; i IRIS Adlershof, Humboldt-Universität zu Berlin, Zum Großen Windkanal 2, 12489 Berlin, Germany; j Department of Molecular Biology, Cell Biology, and Biochemistry, Brown University, 70 Ship Street, Providence, Rhode Island 02912, United States

## Abstract

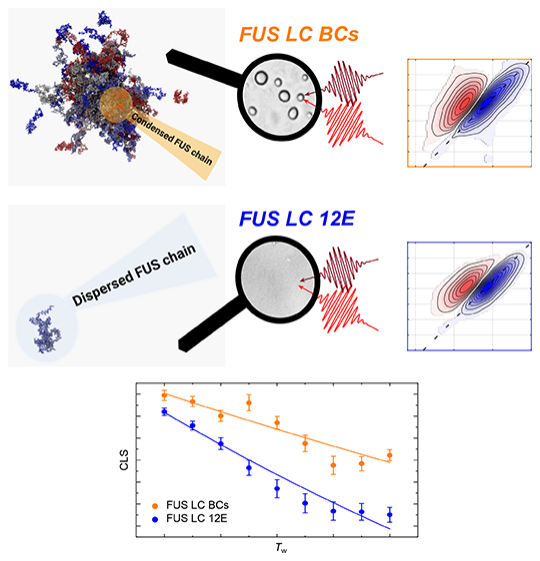

Formation of liquid condensates plays a critical role in biology via localization of different components or via altered hydrodynamic transport, yet the hydrogen-bonding environment within condensates, pivotal for solvation, has remained elusive. We explore the hydrogen-bond dynamics within condensates formed by the low-complexity domain of the fused in sarcoma protein. Probing the hydrogen-bond dynamics sensed by condensate proteins using two-dimensional infrared spectroscopy of the protein amide I vibrations, we find that frequency–frequency correlations of the amide I vibration decay on a picosecond time scale. Interestingly, these dynamics are markedly slower for proteins in the condensate than in a homogeneous protein solution, indicative of different hydration dynamics. All-atom molecular dynamics simulations confirm that lifetimes of hydrogen-bonds between water and the protein are longer in the condensates than in the protein in solution. Altered hydrogen-bonding dynamics may contribute to unique solvation and reaction dynamics in such condensates.

Aqueous solutions of intrinsically disordered proteins can undergo liquid phase separation (LLPS).^1−6^ Proteins that undergo in vitro LLPS condense into protein-rich liquid droplets in which proteins (and other molecules) can diffuse and exchange across the droplet boundaries. These droplets coexist with a continuous, dilute phase that is protein-depleted. Recently, such liquid protein droplets have been termed biomolecular condensates (BCs).^3,7^ The formation of BCs is believed to be critical for regulating cellular organization and biochemistry.^3^ Such regulation can occur due to the enhanced or decreased solubility of reactants in the droplets, which allows steering of biochemical processes. Additionally, the highly crowded environment within the BCs slows diffusive transport, which also impacts biochemistry and may help maintain macroscopic concentration gradients.^3,8−10^

The formation of BCs relies on the subtle balance between protein–protein and water–protein interactions.^11^ As such, the interactions between proteins and water (i.e., hydration), together with hydrogen-bonds (H-bonds) within the BCs, have been proposed to be critical in the condensation process,^2,11−15^ to largely determine the macroscopic and microscopic dynamics,^3,13,16^ and to preserve fluidity and prevent protein aggregation,^17^ pertinent to neurodegenerative diseases. Moreover, H-bonding and hydration also dictate the solvation environment within the BCs, which provides a unique control mechanism for molecular partitioning within the BC. Microscopically, the high concentration of macromolecules in the droplets and changes in the inherent dynamics of the macromolecular matrix can also affect chemical reactivity.^16,18^ Although H-bonding and hydration critically affect biological activity,^19−21^ the intrinsic H-bond dynamics are largely unexplored.^22^

The RNA-binding protein FUS (fused in sarcoma) is a well-studied protein that is known to self-assemble into stress granules in cells and forms inclusions in amyotrophic lateral sclerosis and other neurodegenerative diseases.^3^ The predominant driving force for such self-assembly is the low-complexity (LC) domain of FUS (SYGQ-rich sequence), which is intrinsically disordered.^6^ FUS LC condensation can be triggered by various factors, including pH, temperature, ionic strength, or molecular crowding,^6,10,14,16,23^ which makes FUS LC an ideal model for studying LLPS. Previous studies have shown that these condensates contain a high concentration of FUS LC (∼40% by weight), and nuclear magnetic resonance (NMR) experiments have indicated that FUS LC does not acquire secondary structure upon condensation.^2^ LLPS is suggested to be driven by a manifold of protein–protein interaction motifs with nearly all peptides being involved in the dynamic stabilization of the BCs. The limited change in the secondary structure of FUS LC upon LLPS into droplets deduced from NMR experiments is supported by the Raman vibration bands of FUS, which show only minor changes of the band shape upon phase separation.^2^

The properties of BCs of FUS have been intensively studied. Previous experimental studies established the composition of the BCs, explored the slow conformational dynamics of FUS LC in the BC,^1^ and explored their rheological properties pertinent to the transport of larger objects within the BCs.^24^ On smaller length scales, molecular dynamics simulations have shown that the transport of water and ions is also slowed within the BCs, while the proteins retain their flexibility.^25^ As such, diffusive molecular mobility within BCs is firmly established to be retarded, yet this retardation cannot be extrapolated to the smallest molecular length scales, such as H-bond dynamics, as these H-bond dynamics can be decoupled from macroscopic transport properties. For instance, water dynamics in polyacrylamide hydrogels have been shown to be independent of polymerization (i.e., viscosity),^26^ and in highly crowded cellular environments, a large fraction of water exhibits bulk-like dynamics.^27^ Such inherent molecular-level H-bond dynamics underlie the hydration of proteins^17^ and are pivotal for chemical reactivity.^19,28^ Although experiments have pointed to the importance of hydration in the formation of FUS condensates,^2,29^ the effect of BC formation on hydration, specifically possible differences in the condensate and in the dilute phase, has not been addressed.

Here, we investigate the hydration dynamics in FUS LC BCs. To interrogate the molecular-level environment of FUS LC in BCs, we use the protein’s backbone amide I vibrational mode (C=O of the peptide bond with a high transition dipole moment^30^), which is a ubiquitous, endegenous reporter of the protein’s conformational dynamics and the local H-bonding environment.^28,31^ We use two-dimensional (2D) infrared (IR) spectroscopy^32,33^ to interrogate the spectral dynamics of FUS’s amide groups. Our results show that the short-time (picosecond) decay of frequency correlations of the amide I band is ∼2 times slower for FUS LC within BCs than for FUS LC in a (not phase-separated) homogeneous solution. Molecular dynamics (MD) simulations show that these vastly different dynamics on picosecond time scales can be traced to slowed H-bonding dynamics in the hydration shell of FUS.

To explore the dynamics within BCs, we investigated the low-complexity domain of FUS (FUS LC) using IR spectroscopy. We compare phase-separated FUS LC at approximately neutral pH 7.4 values (denoted FUS LC BCs) to FUS LC at pH 11, which does not phase-separate due to tyrosine deprotonation.^6^ We also examine a phosphomimetic mutant of FUS LC, FUS LC 12E, at pH 7.4 that remains fully homogeneous in solution, i.e., does not undergo LLPS to form droplets, even at millimolar concentrations.^10^ FUS LC self-assembles into micrometer-sized BCs at neutral pH values (Figure 1a), as expected, at a protein concentration of ∼300 μM. Conversely, FUS LC 12E remains homogeneous even at a protein concentration of ∼400 μM (Figure 1b).

**Figure 1:**
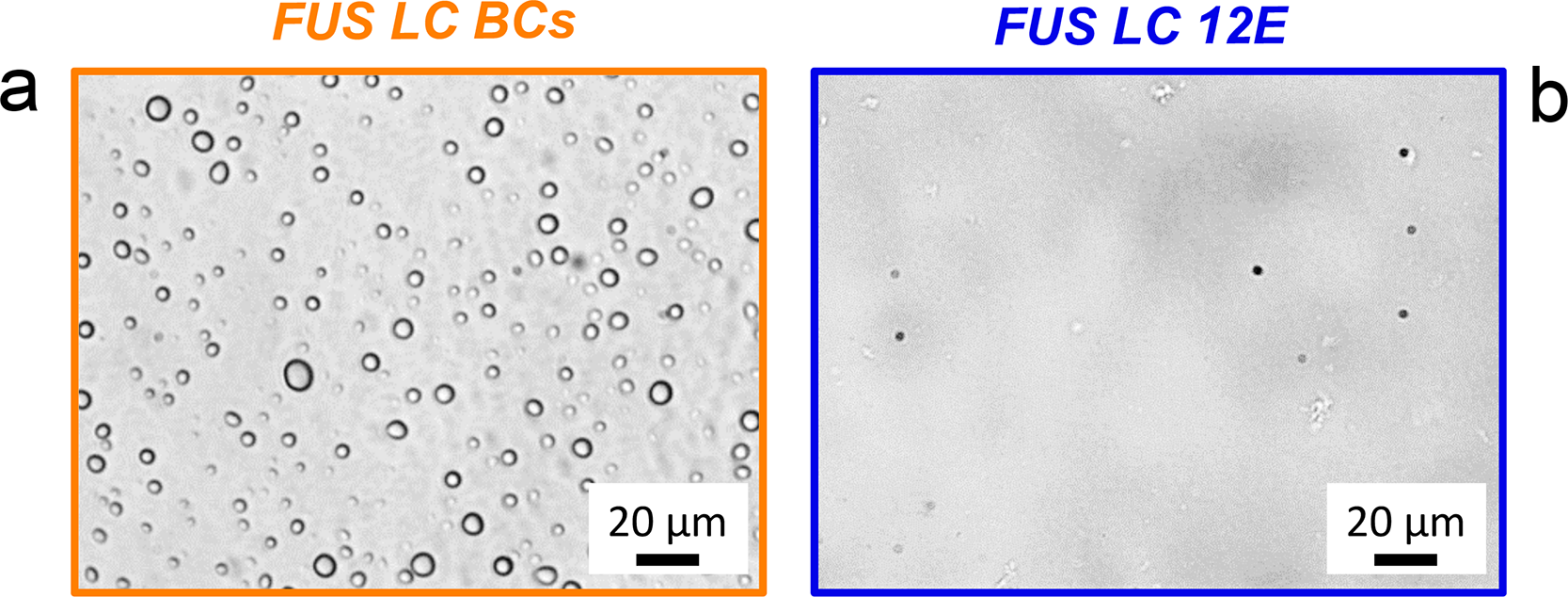
Phase contrast microscopy images of (a) FUS LC BCs with a diameter of 2–10 μm and (b) homogeneous FUS LC 12E. Images were taken after 2D IR experiments. No droplets can be observed in panel b (round species in panel b are imaging artifacts).

The amide I mode of proteins (predominantly the C=O stretching mode of the protein backbone) is an excellent reporter for both changes in protein secondary structure and changes in the protein’s environment.^34^ Thus, to explore spectral changes upon LLPS, we investigate FUS LC’s amide I vibrational band for the homogeneous and condensed forms.^34−38^Figure 2a shows the linear Fourier transform IR spectrum of FUS LC BCs (pH 7.4, orange), FUS LC 12E (pH 7.4, blue), and FUS LC at pH 11 (green) in D_2_O. The spectra were corrected for their solvent background and normalized to the amide I vibration at ∼1640 cm^–1^.

**Figure 2:**
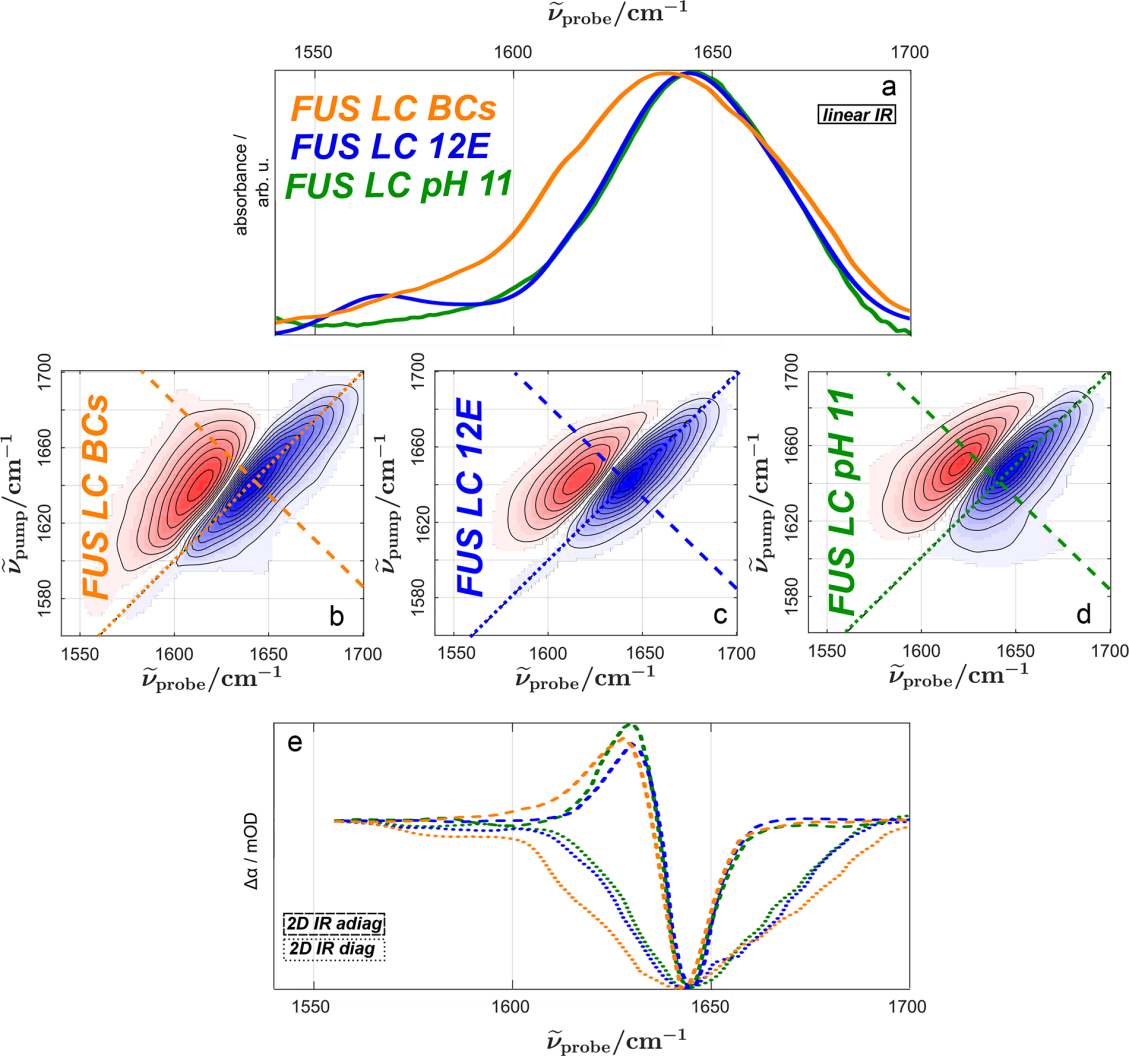
(a) Linear IR spectra of FUS LC BCs (orange), FUS LC 12E (pH 7.4, blue), and FUS LC (pH 11, green). The concentrations for FUS LC (pH 11) and FUS 12E were ∼0.4 mM. All spectra were corrected for their solvent background and normalized to the absorption of the amide I mode at ∼1640 cm^–1^ (see Supplementary Figure 2). The linear spectrum of FUS LC BCs is slightly red-shifted and broader compared to those of FUS LC 12E and FUS LC pH 11. 2D IR spectra at a 0 fs waiting time for (b) FUS LC BCs, (c) FUS LC 12E, and (d) FUS LC pH 11. The dotted lines represent the diagonals for which pump frequency equals probe frequency; the dashed line represents an antidiagonal at 1645 cm^–1^. (e) Diagonal and antidiagonal cuts through the 2D IR spectra as displayed in panels b–d. Antidiagonal cuts at different frequencies are shown in Supplementary Figure 4. The FUS LC BCs sample shows a broader amide I mode compared to those of the FUS LC 12E and FUS LC pH 11 samples, suggesting that the local environment of the peptide bond is more heterogeneous in the droplets.

The amide I band in the IR spectra for FUS LC BCs, FUS LC 12E, and FUS LC at pH 11 is rather broad. For all samples, the amide I band is centered at ∼1640 cm^–1^ (Figure 2a). However, some subtle differences in the band shape for homogeneous (FUS LC 12E and FUS LC pH 11) and phase-separated (FUS LC BCs) proteins can be seen. In the condensed state, the amide I band of FUS LC is somewhat broader and exhibits weak shoulders at ∼1580 and ∼1620 cm^–1^ (Figure 2a), compared to the amide I peak of FUS LC 12E and FUS LC pH 11. The shoulder at ∼1620 cm^–1^ has been related to protein aggregation,^39^ and its contribution is slightly enhanced upon aging of the condensates (see Supplementary Figures 1 and 2). As such, the broadened IR spectra indicate that the amide backbone of FUS senses a broader distribution of microenvironments (inhomogeneous broadening) within the BCs as compared to the homogeneously dissolved protein, and the slight red-shift may point toward stronger H-bonds of the amide backbone to water and/or proteins. Infrared spectra, calculated on the basis of all-atom MD simulations (see below), support the notion that these spectral changes primarily originate from a different hydrogen-bonding environment of the protein in the BCs compared to that of the homogeneously dissolved protein (see Supplementary Figure 3).

The linear vibrational spectra, which reflect the structure and environment of the protein on a subpicosecond time scale, indicate slight differences in the vibrational structure of FUS LC in its homogeneous and condensed states. Conversely, NMR experiments,^2^ sensitive to the average structure on a millisecond time scale, have suggested no significant conformational changes in FUS LC upon phase separation. The time mismatch of these techniques raises the question of how long-lived the structural changes detected with vibrational spectroscopies are.

To address this question, we perform 2D IR spectroscopy experiments on the amide I band.^33^ In a typical 2D IR experiment, vibrational modes are excited with an intense femtosecond IR laser pulse(s), and the response of the excited sample is probed with an infrared probe pulse. This response is determined as a function of excitation frequency. Thus, the response of the system to a specific pump frequency can be detected, which allows for an experimental determination of the frequency–frequency correlations (FFCs) for molecular-level oscillators. Vibrational dynamics can be interrogated by delaying the probing pulse by waiting time *T*_w_ relative to the excitation pulse(s).^33^

Panels b–d of Figure 2 show the 2D IR spectra for condensed FUS LC BCs (Figure 2b) and for homogeneous solvated FUS LC 12E (Figure 2c) and FUS LC pH 11 (Figure 2d) at a *T*_w_ of 0 fs. All spectra display the typical 2D IR features of an inhomogeneously broadened band, a ground state bleach (blue shaded areas) at the excitation frequency (where ν_pump_ = ν_probe_), and a red-shifted (ν_pump_ > ν_probe_) excited state absorption (1 → 2 transition) of the anharmonic oscillators (red shaded areas). The homogeneous line width, which can be assessed from the antidiagonal cut of the 2D spectra (dashed lines in Figure 2b–d), is comparable at 1645 cm^–1^ for the condensed and homogeneous form of FUS LC (dashed lines in Figure 2e; see also Supplementary Figure 4). Conversely, the diagonal cuts (dotted lines in Figure 2e) resemble linear IR spectra. Given the different sensitivities of the linear (squared transition dipole) and 2D IR [fourth power of the transition dipole moment (see Supplementary Figure 5)] spectroscopy, the similarity of the squared linear spectra and the diagonal cuts indicates that the distribution of transition dipoles is similar for FUS LC in the homogeneous and condensed phases.^40^ The broadening of the amide I band upon phase separation is reflected in both the linear and the diagonal cuts of 2D IR spectra. Together, these results suggest that upon BC formation, the intrinsic properties of the amide I mode [e.g., the homogeneous line widths (Figure 2d, dashed line; see also Supplementary Figure 4)] are unaffected. However, the distribution of microenvironments of the amide groups is somewhat altered [inhomogeneous line width, diagonal cut (Figure 2d, dotted line)]. In general, the resonance frequency of an amide mode is sensitive to the H-bonding environment and concomitantly to the secondary structure of a protein.^31,34,41,42^ As such, the enhanced spectral inhomogeneity of FUS LC in the droplets may stem from different protein conformations, resulting in different distributions of inter- and intramolecular protein interactions.^31,43^ However, due to the reduced volume density of solvating water molecules, a broader distribution of the protein hydration motifs, which in turn alters the amide I frequency,^44^ may also give rise to spectral inhomogeneity.

To further explore the source of the spectral inhomogeneity of the amide I band, we explore the vibrational dynamics of the amide mode of FUS by analyzing the waiting time-resolved 2D IR spectra for FUS LC 12E, FUS LC BCs, and FUS LC pH 11 in Figure 3 because the conformational dynamics and hydration dynamics occur on distinct time scales. As common to time-resolved vibrational spectra, the magnitudes of the transient signals decay with an increase in waiting time (e.g., from a maximum normalized bleaching signal in Figure 3a to 25% of the original signal in Figure 3d). This decay is due to relaxation of the excited state population to the vibrational ground state (τ_VER_). To quantify this relaxation, we fit a single-exponential decay [*A*_0_ exp(−*T*_w_/τ_VER_) + *y*_0_] to the integrated peak volumes (see Supplementary Figure 8). From such fits, we find very similar vibrational lifetimes (τ_VER_) of 0.54 ± 0.03 ps for FUS LC 12E, 0.55 ± 0.03 ps for FUS LC BCs, and 0.48 ± 0.02 ps for FUS LC pH 11, which are close to the relaxation of the amide group of the dipeptide alanyl-alanine in D_2_O (0.7 ps).^45^ In general, the value of τ_VER_ is in line with what has been found for other proteins (∼1 ps^46,47^ or less^31,37^) and for the isolated amide moiety of *N*-methylacetamide (0.5 ps).^48^ As such, in line with energy transfer from excited amide groups to the manifold of thermally accessible states being rather insensitive to the primary structure of proteins, we find that condensation also does not alter these dynamics.

**Figure 3:**
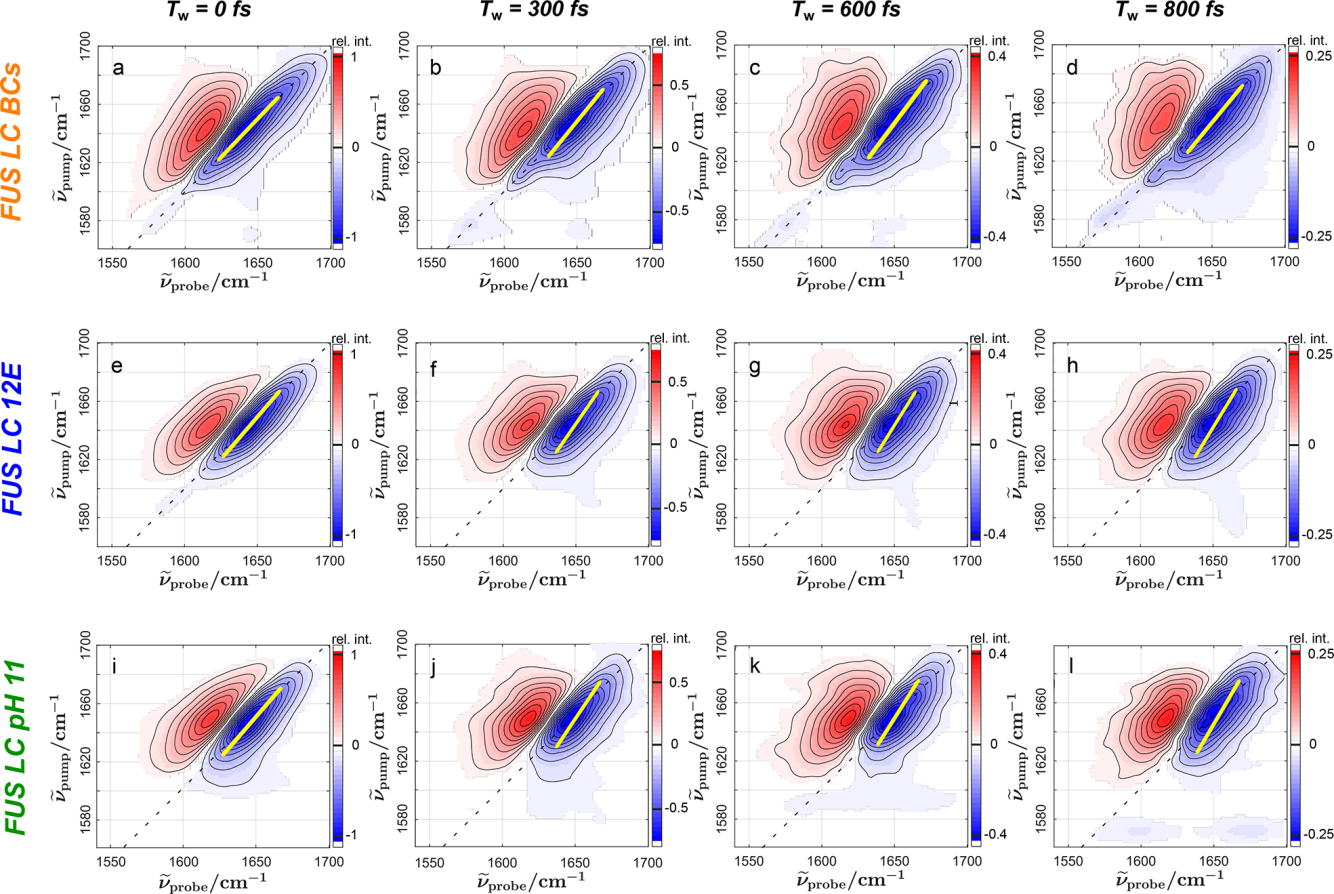
2D IR spectra of FUS LC BCs (top row, a–d), FUS LC 12E (center row, e–h), and FUS LC pH 11 (bottom row, i–l), with increasing waiting time *T*_w_ (from left to right). Yellow lines show the center line slope (CLS) of the ground state bleach (blue). With an increasing waiting time *T*_w_, the CLS, which is almost parallel to the diagonal when *T*_w_ = 0 fs, becomes more parallel to the pump axis. This loss of FFC is more pronounced for FUS LC 12E and pH 11 than for FUS LC BCs; spectral diffusion occurs faster for the homogeneous FUS LC than for the droplets.

Despite the similar vibrational energy relaxation of FUS LC in the homogeneous and condensed phases, we find marked differences in the decay of the FFCs: the temporal variation of the instantaneous frequency of the amide I modes. The FFC can be qualitatively evaluated by elongation of the signals along the diagonal in the 2D spectra. For all three samples, the 2D IR peaks at a *T*_w_ of 0 fs are markedly elongated along the diagonal due to inhomogeneous broadening of the amide I mode. These FFCs are less pronounced at longer waiting times (e.g., *T*_w_ = 800 fs in Figure 3); the peaks appear more vertical. However, for BCs, the ground state bleaching signal remains somewhat elongated along the diagonal (Figure 3d), while for FUS LC 12E and FUS LC at pH 11 solutions, the peaks have already become more vertical (Figure 3h,l). Thus, the detected probe signals show weaker correlation to the excitation frequency at a *T*_w_ of 800 fs for the homogeneous protein solutions compared to the condensate state.

Such FFCs are commonly quantified using the center line slope (CLS): the inverse slope of a straight line fitted through the minima of the transient signals (in the ground state bleach) for a given pump frequency (yellow lines in Figure 3). For a perfectly direct correlation between excitation and detection frequencies, the CLS = 1, whereas the CLS = 0 if the detected response is independent of excitation frequency (i.e., the line is parallel to the pump axis). We show these CLS values as a function of waiting time *T*_w_ in Figure 4. This figure demonstrates that the initial CLS is already somewhat higher for the condensate than for the homogeneous FUS LC from either FUS 12E or FUS LC at pH 11, indicating an enhanced amide I mode spectral inhomogeneity in the condensate. Incidentally, we also note that the starting value for the CLS is higher in FUS 12E (at pH 7.4) than in FUS LC at pH 11, suggesting distinct amide I spectral inhomogeneity under these conditions where FUS is homogeneous, consistent with the disordered nature of the protein and its malleability in different solution environments.^49^ We note that alternatively considering the nodal line slope gives similar results (Supplementary Figure 8). In addition to the differing instantaneous (*T*_w_ = 0) inhomogeneity, the spectral dynamics differ markedly for the different protein states. Within the experimentally accessible time window of ∼800 fs, the CLS value decays by ∼20% for homogeneous FUS LC (FUS LC 12E and FUS LC at pH 11), while in BCs, the CLS value decreases by ∼10%, which suggests 2-fold slower dynamics of the amide mode in the condensed phase as compared to the homogeneous FUS LC. To quantify the decay dynamics, we fit an exponential decay [CLS(*T*_w_) = CLS_0_ exp(−*T*_w_/τ_specdiff_), solid lines in Figure 4] to determine the decay times τ_specdiff_, assuming a simple single-exponential decay. Because the experimental CLS does not decay fully within 800 fs, it may exhibit decay dynamics with more than one time scale, though the short vibrational lifetime poses limitations for more sophisticated models.^50^ Nevertheless, these single-exponential fits appear to describe the experimental data well (Figure 4), and the differences in the spectral diffusion dynamics between both samples can be assessed. From these fits, we find the diffusion time τ_specdiff_ of 2.9 ± 0.2 ps for FUS LC 12E (2.9 ± 0.3 ps for FUS LC at pH 11) to be almost 2 times faster than the value for FUS LC BCs (τ_specdiff_ = 5.5 ± 0.7 ps). The fits confirm the ∼2-fold slower spectral diffusion dynamics of FUS LC in the condensed droplets, relative to that of the homogeneous solution of FUS LC. The decays of the CLS may result from fluctuations of the protein (conformational dynamics) and/or of its environment (H-bonding with water in the proteins’ hydration shell) because both can affect the instantaneous amide I frequencies.^28,31,37,51^ While NMR relaxation rates have indicated that the pico- to nanosecond dynamics of the backbone are slowed in the condensates,^6^ we find marked differences already in the subpicosecond spectral dynamics for FUS LC in the condensed and homogeneous environments. Such subpicosecond dynamics may be affected by different protein structures, such as a sub-ensemble of aggregate proteins that has been associated with the shoulder at 1620 cm^–1^; however, the observed deceleration is insensitive to the frequency range of the CLS analysis (see Supplementary Figure 7). Alternatively, distinct energy transfer dynamics may affect spectral dynamics,^33^ yet the similar vibrational energy relaxation time scales in all three samples suggest changes in energy dynamics are unlikely (see Supplementary Figure 5). Moreover, the observed picosecond time scales are typical for H-bond dynamics, and H-bond fluctuations and H-bond formation/dissociation events can lead to a loss of FFCs as the amide resonance frequency is sensitive to hydration.^28^ As such, we hypothesize that the substantially different dynamics observed in the CLS decays (Figure 4) are largely due to a deceleration of the hydration dynamics of FUS LC within the BCs.

**Figure 4:**
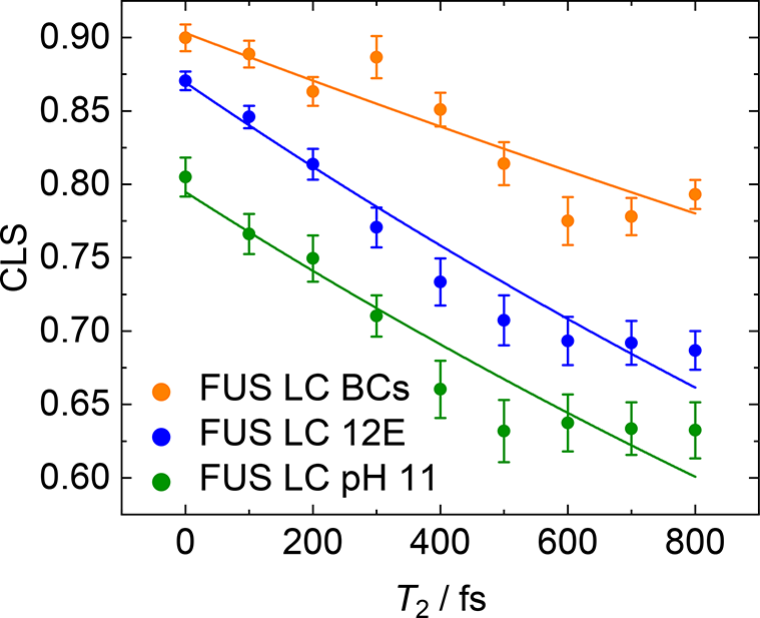
Waiting time (*T*_w_)-dependent CLS of FUS LC BCs (orange), FUS LC 12E (blue), and FUS LC pH 11 (green). CLS of BCs shows a larger value for the instantaneous inhomogeneous broadening [CLS(*T*_w_ = 0 fs)] compared to homogeneous FUS LC in solution. In addition, the CLS of BCs decays slower than that of FUS LC in homogeneous solutions. Solid lines represent single-exponential fits. For all three samples, the same frequency range for the determination of CLS was used (1618–1685 cm^–1^). Even a drastic variation of this frequency range does not alter these trends (see Supplementary Figure 6 and Supporting Information Table 2). Analysis of the nodal line slope confirms the trends obtained from the CLS analysis (Supplementary Figure 7).

To test this hypothesis, we compare our experimental findings to dynamics extracted from fully atomistic MD simulations of a FUS LC droplet in water.^52^ The system contains 134 FUS LC chains in an ∼50 nm cubic box, solvated with >4 million water molecules at a salt (NaCl) concentration of ∼150 mM. The initial configuration for the droplet was obtained from previously performed coarse-grained simulations using the MARTINI model.^53^ After backmapping to atomistic resolution, we equilibrated the system for ∼20 ns to relax the system according to the atomistic model.

To computationally assess the hydration dynamics, we analyze the H-bond lifetime between the amide (C=O) groups of protein chains and water molecules. We then compare the interaction dynamics of FUS LC in the condensed and solvated phases by computing the survival function, i.e., the time autocorrelation function (eq 1):^54^
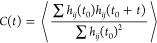
1where *h*_*ij*_(*t*) = 1 if there is a H-bond between the amide oxygen of residue *i* and any hydrogen of water molecule *j* at time *t* and 0 otherwise (see Supplementary Figure 9). The angular brackets denote the averaging over multiple starting points of the trajectory. For detailed information about the calculation of the time autocorrelation function, see the computational methods.

We classify the proteins as either in solution or in the condensate using a simple algorithm that groups proteins together in a cluster if any of their atoms are within 0.3 nm of any atom of another protein (Figure 5a). The correlation functions for water molecules and oxygen atoms on C=O groups for all amino acids in the FUS LC for different states are shown in Figure 5b. These results show that the overall hydration dynamics are qualitatively similar for FUS LC in the condensed and dilute phases. A rapid initial decay of *C*(*t*) is followed by a somewhat slower decay of the H-bond correlation function. However, as already apparent from the raw data at *t* > 0.5 ps, *C*(*t*) decays markedly slower for FUS LC in the BCs than for dilute FUS LC, evidencing longer protein–water H-bond lifetimes in the BCs.

**Figure 5:**
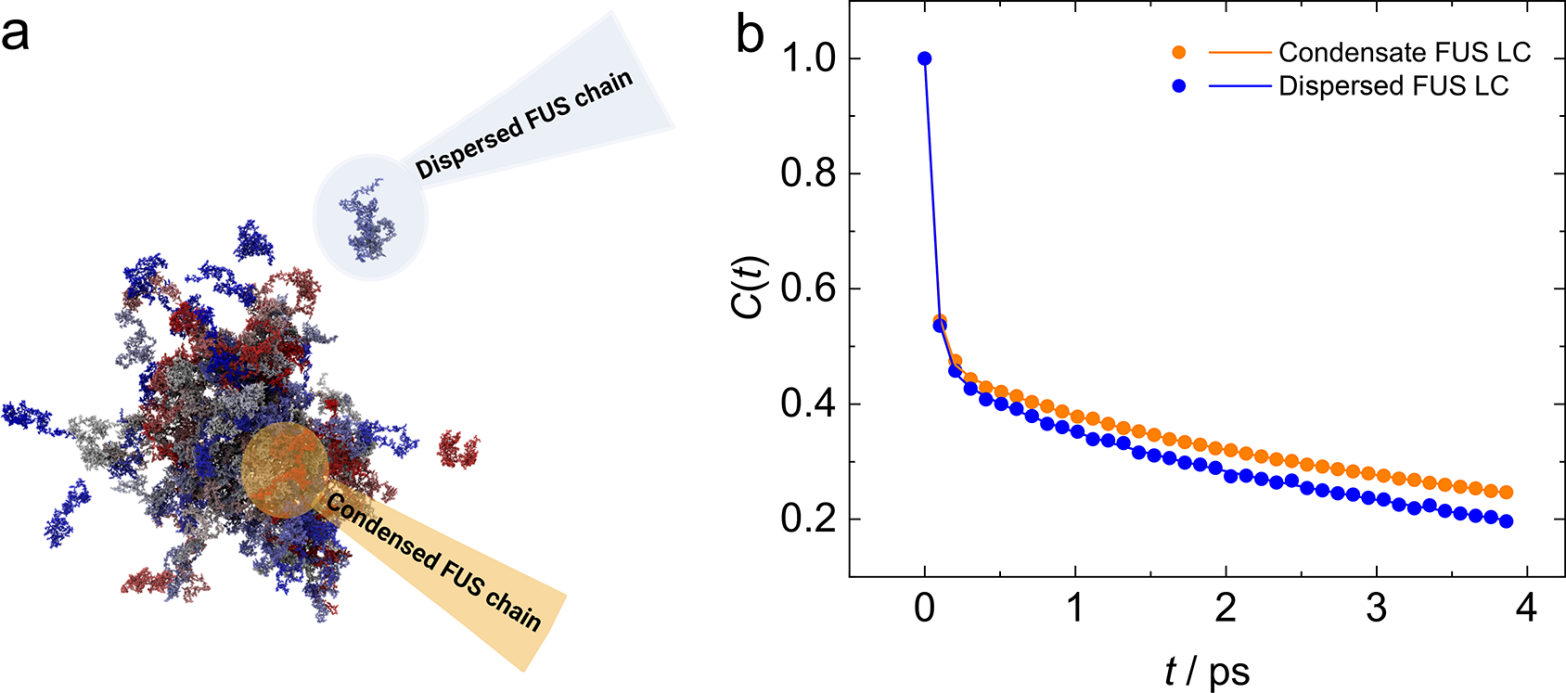
(a) Illustration of the classification of individual FUS protein chains within the atomistic simulations. A simple clustering analysis was performed to identify constituents of the protein droplet as groups of chains in close contact with one another. The remaining protein chains are classified as proteins in the dispersed phase. (b) Autocorrelation function of H-bonds between the carbonyl oxygen and water for FUS LC BC (orange) and FUS LC dilute (blue) showing the slower hydration dynamics in the condensed phase. Solid lines show the triple-exponential fit, and the fit parameters are listed in Table 1.

To quantify these dynamics, we fit a triple-exponential function with three time constants to the data (Supporting Information). The fastest dynamics (time constant of ∼50 fs) are comparable for both protein states, in solution and in the condensate. These can be linked to fast water dynamics, such as librational dynamics of H-bonds of water, but could also arise due to numerical effects in the analysis. However, the slower dynamics (time constants of approximately 0.5–0.8 and 5–7 ps) are consistently slower in the condensate than for FUS LC in solution (see Supplementary Figure 10). Both time scales are consistent with the 2D IR experimental results and are typical time scales of H-bond lifetimes. This observation of two distinct time scales in the simulations (0.5–0.8 and 5–7 ps) may point to heterogeneous water accessibilities of the amide backbone, yet the longer time scales (5–7 ps) could also be related to H-bond breaking triggered by the motion of multiple heavy atoms, such as side chain rotation. Nevertheless, this analysis confirms that the lifetimes of H-bonds between water and the amide C=O group are markedly longer (by ∼40%) for FUS LC in the condensates than for dilute FUS LC. Given that the hydration state of the amide C=O is intimately related to the amide I resonance frequency,^55^ the increased H-bond lifetimes in the condensates observed in the simulations can largely explain the slower decay of FFCs as experimentally observed in the 2D IR experiments.

**Table 1:** Fit Parameters of the Triple-Exponential Fit to the Autocorrelation Function for Hydrogen Bonds between FUSs’ Carbonyl Groups and Water^a^

	τ (ps)	τ_1_ (ps)	τ_2_ (ps)	τ_3_ (fs)	*A*_1_	*A*_2_	*A*_3_
condensed	3.2 ± 0.2	7.5 ± 0.3	0.77 ± 0.13	50.0 ± 0.1	0.41 ± 0.01	0.07 ± 0.01	0.52 ± 0.02
in solution	2.3 ± 0.2	5.4 ± 0.2	0.58 ± 0.15	50.0 ± 0.2	0.41 ± 0.01	0.07 ± 0.01	0.52 ± 0.02

a *A_j_* and τ_*j*_ are the amplitudes and time constants, respectively, of the three exponentials. τ is the amplitude-weighted average time constant (see the Supporting Information). Errors were obtained from the diagonal elements of the covariance matrix of the nonlinear fit.

In summary, we probed the effect of LLPS of FUS LC on the vibrational dynamics of the amide I mode, a characteristic vibrational probe for the protein backbone, using time-resolved vibrational spectroscopy. Linear infrared spectra reveal a 12% wider line width for FUS LC in the condensate than in FUS LC at pH 11 or FUS LC 12E at neutral pH, each of which remains fully homogeneous as a solution. Strong FFCs in 2D IR spectra show that the line widths of the linear IR spectra originate from inhomogeneous broadening, indicative of a broader range of microenvironments of the amide backbone of FUS LC in the condensates as compared to the dilute FUS LC. Within the experimentally accessible time window of ∼800 fs, we find the characteristic decay time of the FFCs is 2-fold longer in the condensate than in the dilute protein. The (sub)picosecond time scale of these spectral dynamics suggests that they are intimately related to water dynamics in protein hydration shells, rather than conformational dynamics of the protein giving rise to fluctuations of the amide I frequency. All-atom MD simulations of FUS LC qualitatively support these results; H-bond lifetimes of water molecules hydrating the amide backbone are ∼40% longer in the condensate than in dilute FUS LC.

Our findings show that, in contrast to the case for hydrogels,^26^ macroscopic transport and microscopic hydration dynamics are correlated for FUS. This correlation may be rationalized by considering that the H-bond lifetimes in the hydration shells are closely related to exchange dynamics: as the density of water molecules within the condensates is reduced, H-bond exchange rates within the BCs decrease,^56^ resulting in more persistent hydration shells of FUS and potentially of other solutes in the BCs. As H-bonding is paramount for the solvation of solutes and transition states, the slower hydration dynamics within the condensates may be critical to understanding how rates of biochemical processes, such as enzymatic processes or electron transfer reactions in which the rigidity of the hydration shell is important,^19−21^ can be controlled via LLPS.

## Methods

*Protein Purification*. Unless otherwise stated, all reagents were purchased from Sigma Aldrich.

FUS LC was prepared according to the method of Chatterjee et al.^1^ using Addgene plasmid 127192^2^ with the following minor adaptations to the experimental procedure. (1) The inclusion body solubilization was supported with the aid of a mortar and some additional sonication. (2) Elution was from a Ni-NTA column with increasing imidazole concentrations of ≤400 mM. (3) The solubilizing buffer:tobacco etch virus (TEV) protease mass ratio was increased to 1:5 (or even 1:3 according to the activity of TEV, instead of 1:20). His-cleaved FUS LC was stored at 1–2 mM in 20 mM CAPS buffer (pH 11) at −80 °C. FUS LC protein was either dialyzed into D_2_O CAPS buffer or lyophilized from CAPS buffer and then stored at −80 °C.

FUS LC 12E was produced according to the procedure by Monahan et al. using Addgene plasmid 98654.^10^ In short, bacterial pellets of cells with FUS LC 12E expressed were resuspended in 20 mM sodium phosphate, 300 mM sodium chloride, and 10 mM imidazole (pH 7.4), lysed by ultrasonic digestion, and centrifuged at 18500*g* for 60 min at 4 °C. For purification, the supernatant was loaded on 5 mL of Ni-NTA and eluted with a concentration gradient from 10 to 300 mM imidazole. Subsequently, fractions containing protein were identified via sodium dodecyl sulfate–polyacrylamide gel electrophoresis and diluted with resuspension buffer without imidazole to decrease the imidazole concentration to ∼40 mM. The mixture was then incubated with TEV protease (1:3 TEV:solubilizing buffer) overnight at room temperature. The protein sample was subsequently filtered over a Ni-NTA 5 mL column, resulting in a His tag free protein. All protein-containing fractions were pooled. Consecutively, a buffer exchange to CAPS buffer (pH 11, 20 mM) was performed by dialysis with 6–8 kDa Ready-A-Lyzer (Thermofisher). The samples were concentrated using 3 kDa Amicon filters (first concentrated and then dialyzed). A 280 nm photometry experiment determined the concentration of the protein to be ∼700 μM. The sample was stored at −80 °C.

*Sample Preparation for IR Measurements*. Lyophilized samples were resolubilized in D_2_O. The D_2_O-soluibilized samples were diluted with CAPS buffer prepared in D_2_O to reach a concentration of 700 μM using the absorbance at 280 nm with an extinction coefficient of 35 760 M^–1^ cm^–1^. Next, the pH was adjusted by the addition of phosphate buffer (pD 5.5, 20 mM sodium phosphate, 150 mM NaCl) to a final pD value of 7.5 and a concentration of ∼0.4 mM.

*Fourier Transform Infrared (FT IR) Measurements*. A Bruker Vertex 70 spectrometer was used to record linear FT IR spectra in transmission geometry with a resolution of 4 cm^–1^ at frequencies ranging from 400 to 4000 cm^–1^. To ensure a water vapor free environment during the measurement, dried air was used to purge the sample compartment. The IR measurements were performed in a cell consisting of two CaF_2_ windows (2 mm thickness and 2.54 cm diameter) separated by a 25 μm Teflon spacer. The linear IR spectra were collected immediately before and after the 2D IR measurements (Supplementary Figure 1).

*2D IR Measurements*. A commercial 2D Quick IR (Phasetech Inc.) setup was used to record the 2D spectra of FUS LC. The exact setup is described elsewhere.^45^ Briefly, 800 nm laser pulses (7 W, 35 fs, 1 kHz) from a regenerative amplifier laser (Coherent, Astrella) were used to pump an optical parametric amplifier Topas Prime (Coherent) with a noncollinear difference frequency generation (NDFG) stage to generate pulses at 6000 nm (18 μJ, 400 cm^–1^ full width at half-maximum). The IR pulses are guided into a 2D Quick IR setup (Phasetech), which uses an acousto-optical modulator to generate pump pulse pairs.

Pump frequencies were resolved by recording pump pulses delayed by 0–2555 fs in increments of 35 fs. To reduce the measurement time, a rotating frame at 1400 cm^–1^ was applied. The acquired time-domain data were zero-padded to 128 data points and filtered with a Hamming window before Fourier transformation. Frequency-resolved modulation of the probe pulses by the pump pulse excitation was recorded by dispersing the probe beam onto a 128 × 128 mercury cadmium telluride (MCT) array detector.

*MD Simulations*. The CG condensed conformation was obtained using an adaptation of the MARTINI CG model,^57^ and the trajectory file of the simulation was provided by the authors of this work. The system was then backmapped utilizing CHARMM-GUI^58^ andthe CHARMM-GUI Martini Maker^59^ all-atom converter tool that utilizes the backward.py program.^60^

The all-atom simulated system encompasses 134 chains of the low-complexity region of the FUS protein (FUS LC), placed in an ∼50 nm cubic box and explicitly solvated with 4 222 084 water molecules and 24 202 Na and Cl ions, reaching a salt concentration of ∼150 mM; this resulted in a simulation box containing 17 213 858 atoms.

The simulation was performed with the GROMACS 2019.3 simulation suite,^61,62^ and the a99SB-disp force field^63^ was used to model protein interactions and the four-point a99SB-disp^64^ for water molecules. The equations of motion were solved with a time step of 2 fs with periodic boundaries applied in all directions. All covalently bonded hydrogens were constrained using the P-LINCS^65^ algorithm. The long-range electrostatic interactions were evaluated using particle-mesh Ewald summation,^64^ while the Lennard-Jones and short-range electrostatic interactions were truncated at a cutoff distance of 12 Å.

The system was first energy minimized using the steepest descent algorithm. Subsequently, a series of equilibration simulations with position restraints applied to the heavy atoms of the FUS chains were performed: (1) two 125 ps *NVT* simulations employing a 1 fs time step, the Berendsen thermostat with a coupling constant of 1 ps, and restraint force constants of 1000 and 400 kJ mol^–1^ nm^–2^, (2) a 125 ps *NPT* simulation employing a 1 fs time step, the Berendsen thermostat and barostat with coupling constants of 1 and 5 ps, respectively, and a restraint force constant of 200 kJ mol^–1^ nm^–2^, (3) two 500 ps *NPT* simulations employing a 2 fs time step, the Berendsen thermostat and barostat with coupling constants of 1 and 5 ps, respectively, and restraint force constants of 200 and 100 kJ mol^–1^ nm^–2^, respectively, (4) a 1.5 ns simulation with the same parameters for the thermostat and barometer as step 3 but completely without positional restraints, and (5) a 19 ns equilibration run performed in the *NPT* ensemble at 300 K utilizing the Parrinello–Rahman barostat with a time constant coupling of 2 ps and the Bussi–Donadio–Parrinello velocity rescaling (V-rescale) thermostat with a coupling time constant of 0.1 ps. Finally, a production simulation run of 1 ns with the same parameters that were used for simulation run number 5 was performed. Characterization of each protein as “dispersed” or “condensed” was performed using the analysis function gmx clustzise within the Gromacs simulation package with a cutoff distance of 0.3 nm. The categorization was further corroborated by utilizing the MDAnalysis^2,14^ package to determine that no protein categorized as a member of the dispersed group had any contact within 0.3 nm of any protein categorized as condensed.

*Fit of the H-Bond Correlation Function*. The baseline H-bond population is removed from the autocorrelation function, and the function is fitted to a triple-exponential function with three components.^54^
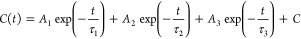
where the pre-exponential factors are subject to the condition
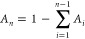


The different τ_*n*_ values refer to each of the representative time scales of the H-bond dynamics around the amide group. τ_3_ is likely related to fast water dynamics, such as librational dynamics of water or hydrogen-bond stretching dynamics, but could also arise due to numerical effects in the analysis. τ_2_ and τ_1_ may be linked to the heterogeneous water accessibility of the amide backbone, yet the longer time scales (5–7 ps) could also be linked to movements of multiple heavy atoms, such as side chain rotation. We then obtain an overall time constant τ, which is the weighted average of the fitting function by integrating the correlation function:
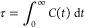


*Calculation of Linear IR Spectra*. The spectral calculations are based on the formalism described in ref (66), later adapted in a FORTRAN90 environment.^67^ Briefly, the diagonal elements of the amide I one-exciton Hamiltonian (i.e., the local modes) are modeled using the linear relationship between hydrogen-bonding strength and amide C=O length, as determined by Cho et al.^28^ The parametrization, with a gas phase frequency (Ω_0_) of 1655 cm^–1^ and a scaling factor with C=O length differentiation from 1.229 Å of 400, has been calibrated to an LKa14 peptide (see ref (68) for details). Furthermore, the diagonal elements upstream of proline residues are red-shifted by 19 cm^–1^, to account for the heavier weight of the ring connected to the nitrogen atom of that backbone amide group. The spectral sensitivity to the conformation further comes through the off-diagonal elements of the Hamiltonian where two different coupling models are employed to estimate the nearest- and non-nearest-neighbor couplings. As the former are dominated by through-bond effects, we model these using a parametrized DFT map of the coupling as a function of the dihedral angles (ϕ and ψ), based on comprehensive quantum chemical calculations on the “glycine dipeptide” (Ac-Gly-NHCH_3_), using the 6-31G+(d) basis set and B3LYP functional.^33,69^ The latter couplings are dominated by through-space effects, which we therefore estimate with the transition dipole coupling model.^70,71^ Subsequently, the delocalized vibrational eigenmodes are determined by diagonalizing the one-exciton Hamiltonian, which is convoluted with Lorentzian line shapes with a half-width at half-maximum of 6 cm^–1^.

## Supporting Information Available

Linear FT IR spectra before and after 2D IR experiments, details about background subtraction from linear FT IR spectra, analysis of simulated FT IR spectra, antidiagonal line widths of 2D IR spectra, comparison of squared linear FT IR spectra and diagonal cuts through 2D IR spectrum, effect of the frequency range on CLS analysis, nodal line slope versus center line slope dynamics, determination of vibrational relaxation lifetime, schematics of amide group hydrogen-bond criteria, and fit stability test for the H-bond correlation function (PDF)
