# Supplementary material for: Liquid–Liquid Phase Separation of the Intrinsically Disordered Domain of the Fused in Sarcoma Protein Results in Substantial Slowing of Hydration Dynamics
Source: J Phys Chem Lett. 2023 Dec 6;14(49):11224–34. doi: 10.1021/acs.jpclett.3c02790 (PMC10726384; doi:10.1021/acs.jpclett.3c02790)
Supplement: Supplementary file 1 — jz3c02790_si_001.pdf [file jz3c02790_si_001.pdf]

SUPPORTING INFORMATION

for

Liquid-Liquid Phase Separation of the Intrinsically Disordered Domain of the Fused in Sarcoma Protein Results in Substantial Slowing of Hydration Dynamics

Carola S. Krevert,[a,+] Daniel Chavez,[b,+] Sayantan Chatterjee,[a,c] Lukas S. Stelzl,[d] Sabine Pütz,[a] Steven J. Roeters,[e,f] Joseph F. Rudzinski,[b,g] Nicolas L. Fawzi,[h] Martin Girard,[b,*] Sapun H. Parekh,[a,c,*] and Johannes Hunger[a,*]

---

[a] *C. S. Krevert, Dr. S. Chatterjee, S. Pütz, Prof. S. H. Parekh; Dr. J. Hunger, Department of Molecular Spectroscopy, Max Planck Institute for Polymer Research, Ackermannweg 10, 55128 Mainz (Germany)*

[b] *D. Chavez, Dr. J. F. Rudzinski, Dr. M. Girard, Department of Polymer Theory, Max Planck Institute for Polymer Research Ackermannweg 10, 55128 Mainz (Germany)*

[c] *Dr. S. Chatterjee, Prof. S. H. Parekh, Department of Biomedical Engineering, The University of Texas at Austin 107 W Dean Keeton Street Stop C0800, Austin, TX 78712 (USA)*

[d] *Dr. L. Stelzl, KOMET 1, Institute of Physics, Johannes Gutenberg University, Staudingerweg 7, 55099 Mainz (Germany) Faculty of Biology, Johannes Gutenberg University Mainz, Gresemundweg 2, 55128 Mainz (Germany) Institute of Molecular Biology (IMB), Ackermannweg 2, 55128 Mainz (Germany)*

[e] *Dr. S. J. Roeters, Department of Chemistry, Aarhus University, Langelandsgade 140, 8000 Aarhus C. (Denmark)*

[f] Dr. S.J. Roeters, Department of Anatomy and Neurosciences, Amsterdam UMC, Vrije Universiteit, De Boelelaan 1108, 1081 HZ, Amsterdam (The Netherlands)

[g] *Dr. J. F. Rudzinski, IRIS Adlershof, Humboldt-Universität zu Berlin, Zum Großen Windkanal 2, 12489 Berlin (Germany)*

[h] *Prof. N. L. Fawzi, Department of Molecular Biology, Cell Biology, and Biochemistry, Brown University 70 Ship Street, Providence, RI 02912 (USA)*

[+] *contributed equally*

[*] *Email: girard01@mpip-mainz.mpg.de ; sparekh@utexas.edu; hunger@mpip-mainz.mpg.de*

Table of Contents

SUPPLEMENTARY FIGURES 3

LINEAR FT IR SPECTRA BEFORE AND AFTER 2D IR MEASUREMENT 3
BACKGROUND SUBTRACTION OF LINEAR FT IR SPECTRA 4
CALCULATED FT IR SPECTRA 5
ANTIDIAGONAL LINE WIDTHS 6
COMPARISON OF SQUARED LINEAR FT IR SPECTRA AND DIAGONAL CUTS THROUGH 2D IR SPECTRUM 7
VARIATION OF FREQUENCY RANGE FOR CLS ANALYSIS 8
NODAL LINE SLOPE VS CENTER LINE SLOPE DYNAMICS 9
DETERMINATION OF VIBRATIONAL RELAXATION LIFETIME 10
DEPICTION OF AMIDE GROUP HYDROGEN BOND 11
FIT STABILITY TEST 11

Supplementary Figures

Linear FT IR spectra before and after 2D IR measurement

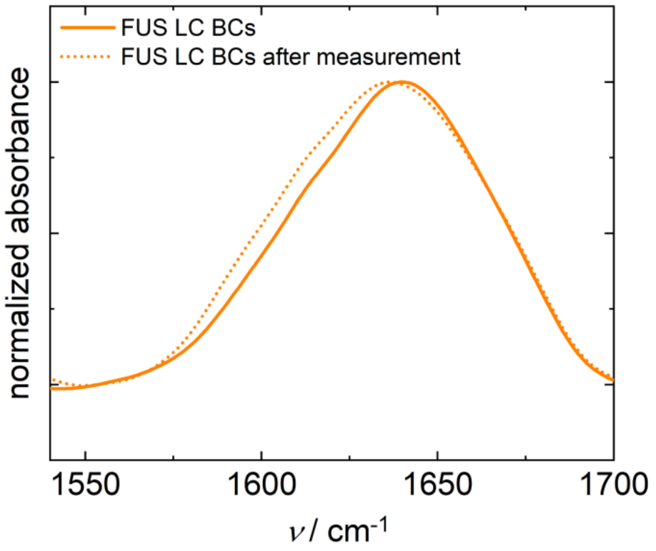

Supplementary Figure 1. Background subtracted linear IR spectrum of FUS LC BCs before (solid) and after (dotted) 2D IR measurement: Upon aging a minor broadening of the amide I band towards lower frequencies is observed.

Background subtraction of linear FT IR spectra

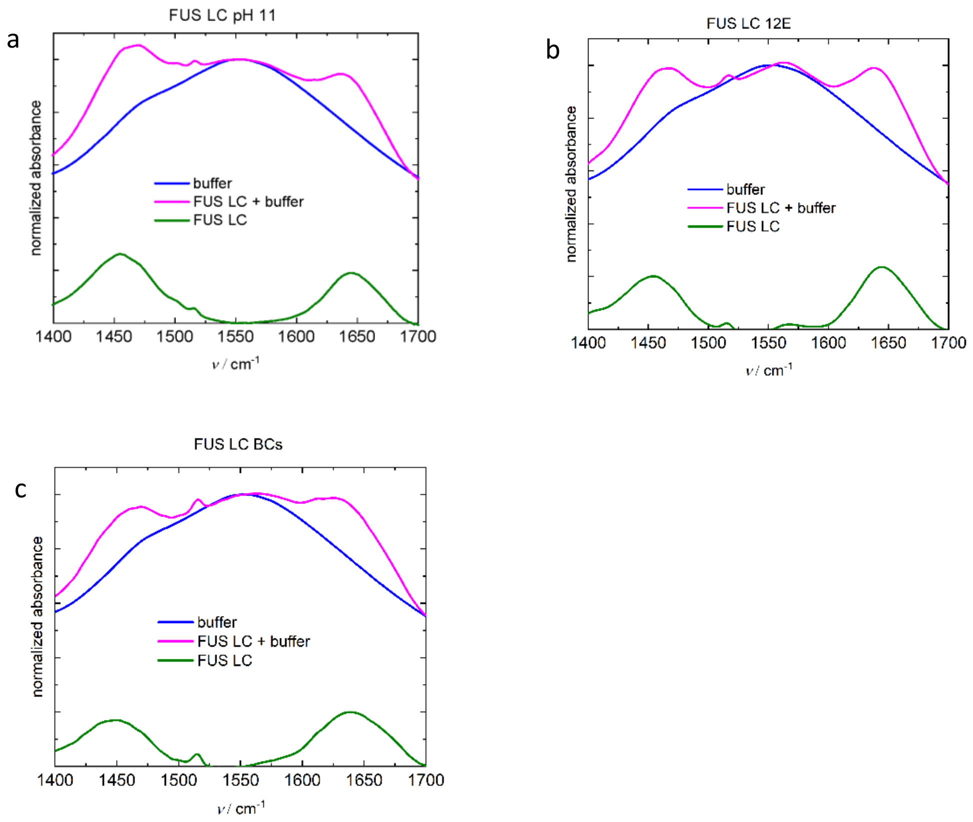

Supplementary Figure 2. Linear IR spectra of FUS LC pH 11 (a), FUS LC 12E (b) and FUS LC BCs. Pink lines show the spectra of FUS LC + buffer solution, blue lines represent the IR spectra of pure buffer solution. The buffer and buffer + FUS LC spectra were normalized to the maximum intensity of the buffer spectrum at 1550 cm^{-1} and subsequently subtracted from each other, resulting in the green difference spectrum. Signals at 1450 cm^{-1} are assigned to the amide II mode; signal at 1510 cm^{-1} represents the deprotonated tyrosine residues of FUS LC residues.

Calculated FT IR spectra

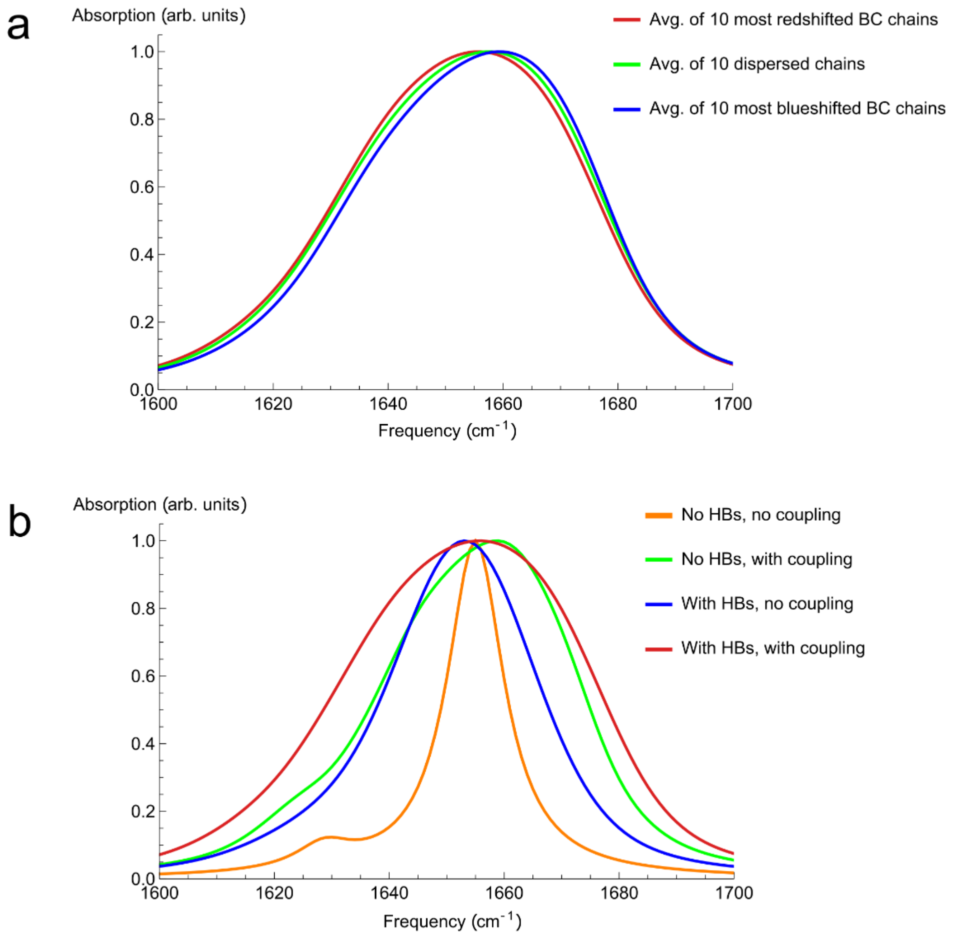

Supplementary Figure 3. Spectral calculations that indicate broad spectral distribution in ensemble of BC chains and that H-bonding effects dominate the observed spectral broadening. a) Within the ensemble of protein chains that reside within the BC, we can find sub-ensembles that are either red- or blue-shifted (depicted by the red and blue traces) with respect to the dispersed chains (depicted by the green trace). The limited degree of red- and blue-shifting could partially be caused by the small number of chains in the droplets, causing many of them to be on the 'condensate surface'. b) Although both coupling and H-bonding cause the spectra to be broadened, relatively strong H-bonding seems to dominate the spectra of the 10 most redshifted FUS-chains. We determine this by turning on/off the different spectral modeling elements that give rise to the spectra, calculated using the one-exciton Hamiltonian approach (see Methods). Without any coupling- or H-bonding-model on, the local modes are fixed to the so-called "gas phase" frequency (set to 1655 cm^{-1}, in accordance with Roeters et al.[1]). Amide groups downstream of proline residues (which contain a heavier ring moiety instead of the proton that other amino acids have at the nitrogen atom) are redshifted by 19 cm^{-1}, in accordance with Roeters et al.[2] Then, when coupling models are turned on, the normal modes broaden as expected, but the red-shifting is mostly a result of the inclusion of the hydrogen-bonding model. Both effects combined lead even to more broadening (also as expected with these one-exciton Hamiltonian calculations).

Antidiagonal line widths of 2D IR spectra

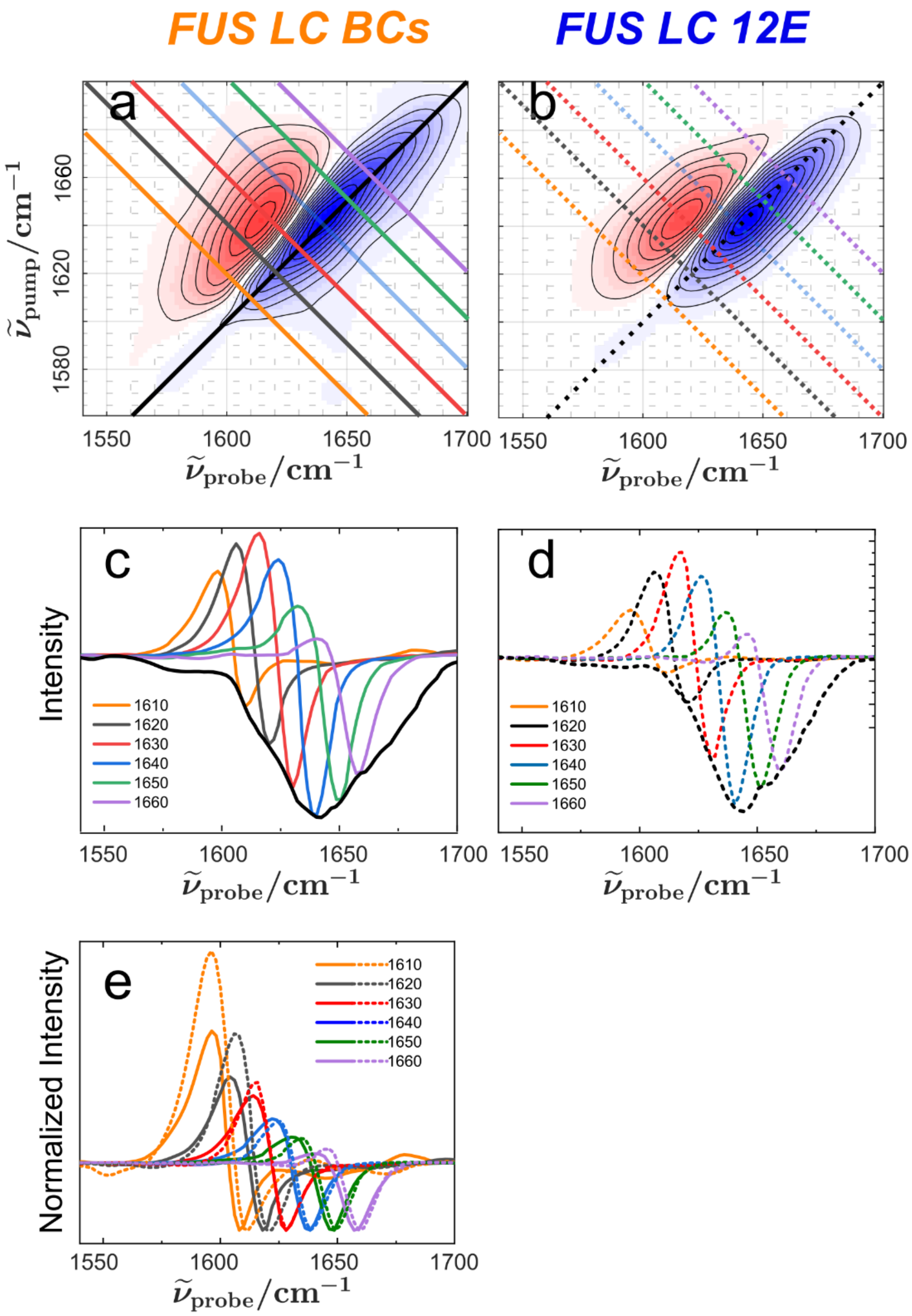

Supplementary Figure 4. 2D IR spectra at 0 fs waiting time of FUS LC BCs (a) and FUS LC 12E (b) with anti-diagonal cuts from 1610 cm^{-1} to 1660 cm^{-1} at increments of 10 cm^{-1} plotted in (c) and (d) for anti-diagonal cuts and diagonal cut (solid black line). Figure e shows the anti-diagonal cuts normalized to the minimum for FUS LC 12E (dashed line) and FUS LC BCs (solid line): The width of the negative signals is approximately the same for all frequencies and samples.

Comparison of squared linear FT IR spectra and diagonal cuts through 2D IR spectrum

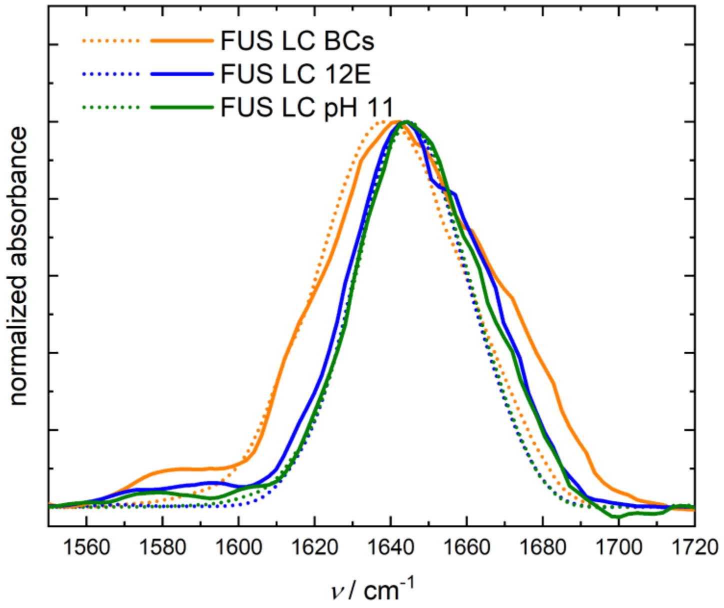

Supplementary Figure 5. Squared linear IR spectra (solid lines) and diagonal cuts of the 2D IR spectra (dotted lines) of FUS LC BCs (orange), FUS LC 12E (blue) and FUS LC pH 11.

Variation of frequency range for CLS analysis

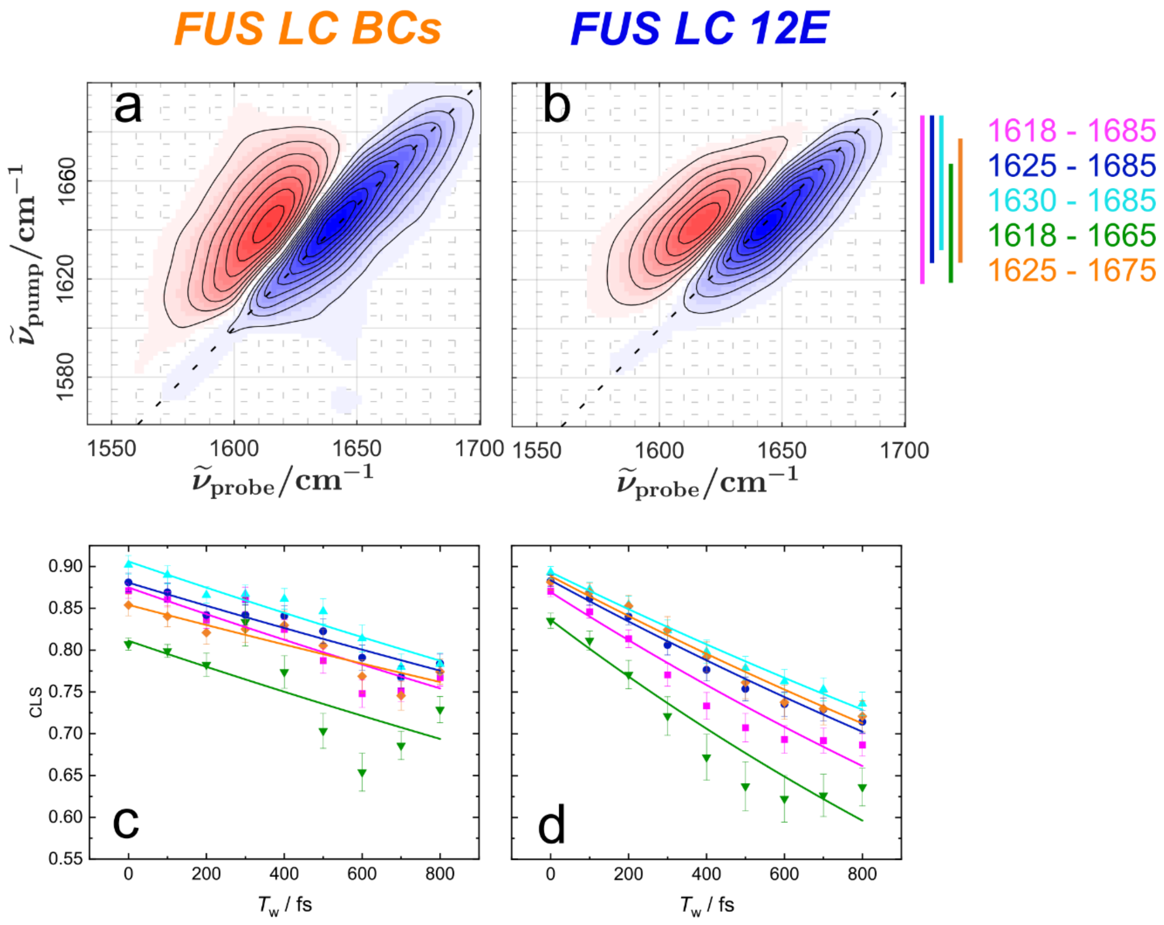

Supplementary Figure 6. 2D IR spectra at 0 fs waiting time of FUS LC BCs (a) and FUS LC 12E (b) with the frequency ranges for the CLS determination (marked as colored bars, right). CLS decay and mono-exponential fit for FUS LC BCs (c) and FUS LC 12E (d) using different frequency ranges for the CLS analysis: The variation of the frequency range for CLS analysis does not alter the qualitative differences between FUS LC BCs and FUS LC 12E and the CLS decays slower for FUS LC BSc throughout (see also Supplementary Table 2).

Supplementary Table 1. Spectral diffusion time ($\tau_{specdiff}$) obtained using mono-exponential fits of the waiting time dependent CLS for FUS LC BCs and FUS LC 12E obtained using different frequency ranges. The ratio between both values is given in the last line: The approximately two times slower decay for FUS LC BCs as compared to FUS LC 12E is independent of the considered frequency ranges.

CLS frequency range / cm^{-1}	1618-1685	1625-1685	1630-1685	1618-1665	1625-1675
$\tau_{specdiff}$ (FUS LC BCs) / ps	5.4 ± 0.8	6.3 ± 0.7	5.7 ± 0.6	5.1 ± 1.1	6.9 ± 1.1
$\tau_{specdiff}$ (FUS LC 12E) / ps	2.9 ± 0.2	3.5 ± 0.1	3.9 ± 0.1	2.4 ± 0.2	3.6 ± 0.2
$\tau_{specdiff}$ (FUS LC BCs) / $\tau_{specdiff}$ (FUS LC 12E)	1.9	1.8	1.5	2.1	1.9

Nodal line slope vs center line slope dynamics

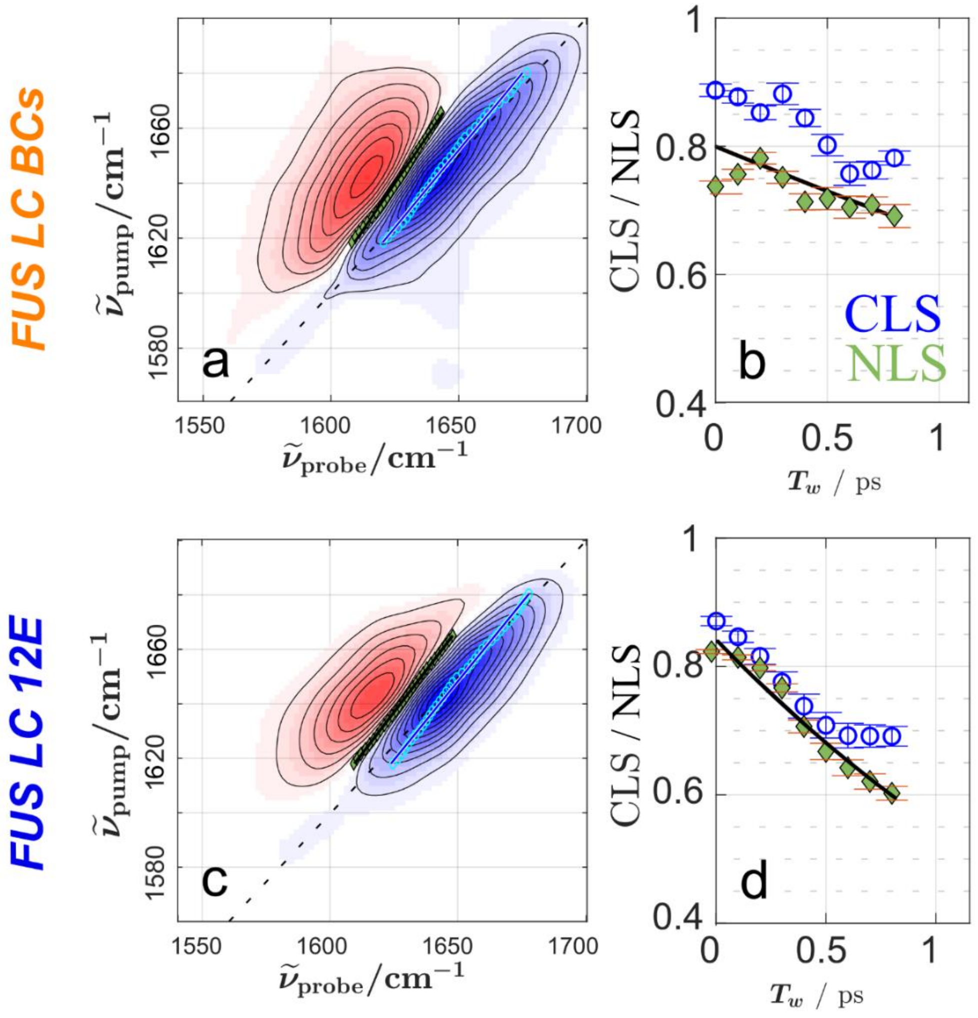

Supplementary Figure 7. 2D IR spectra at 0 fs waiting time (T_w = 0 fs) for FUS LC BCs (a) and FUS LC 12E (c). The blue dots mark the minimum of the ground state bleach of slices parallel to the probe axis in pump a frequency range from 1618 – 1680 cm^{-1} for both spectra; the blue line shows linear fits through these points, where the inverse slope of these fits is the center line slope, CLS. The green diamonds mark the nodal point (zero signal between ground state bleach and excited state absorption, also taken from slices parallel to the probe axis (at pump frequencies ranging from 1618 to 1665 cm^{-1} for both spectra); green line marks the linear fit of these nodal points. The inverse slope of these fits represents the nodal line slope, NLS. Waiting time dependent values for CLS and NLS are shown for FUS LC BCs (b) and FUS LC 12E (d): The CLS and NLS show the same trend for both FUS LC BCs and FUS LC 12E. Time constants of the mono-exponential fit for NLS: $\tau_{specdiff(FUS\ LC\ BCs)}$ = 9.7 ps; $\tau_{specdiff(FUS\ LC\ 12E)}$ = 2.5 ps indicate a marked slow-down of the spectral dynamics ($\tau_{specdiff}$ (FUS LC BCs) / $\tau_{specdiff}$ (FUS LC 12E)) = 3.8).

Determination of vibrational relaxation lifetime

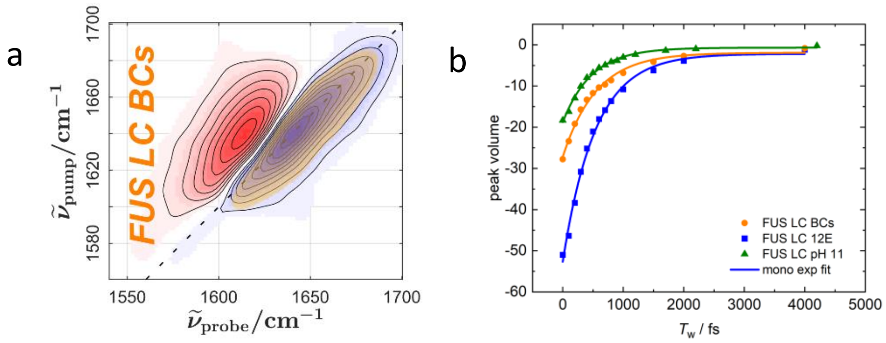

Supplementary Figure 8. a) 2D IR spectrum of FUS LC BCs at 0 fs waiting time (T_w). Orange ellipse represents the integration volume used for determination of the waiting time dependent peak volume to extract the vibrational relaxation lifetime. The same volume has been used to extract the peak volume for FUS LC 12E and FUS LC pH 11, b) Waiting time (T_w) dependent peak volume of the amide I mode of FUS LC BCs (orange), FUS LC 12E (blue), and FUS LC pH 11 (green) from 2D IR spectra. Solid lines are single exponential fits (A_0 exp(-T_w/τ_{VER}) + y_0). Vibrational relaxation lifetime (τ_{VER}) is approximately 0.5 ps for all samples (0.54 ps for FUS LC 12E, 0.55 for FUS LC BCs and 0.48 ps for FUS LC pH 11).

Depiction of amide group hydrogen bond

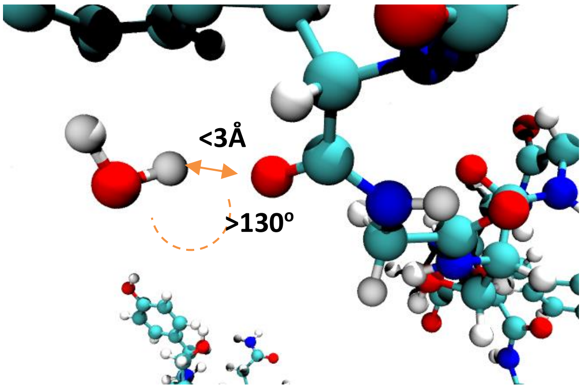

Supplementary Figure 9. Depiction of water molecule forming a hydrogen bond with FUS (for clarity other FUS proteins and water molecules in the vicinity are not shown). A water molecule is considered to form a hydrogen-bond if any hydrogen of the molecule is closer than 3 Å from the oxygen of the amide I and has an angle of more than 130° between the O(water)-H(water)-O(FUS amide).

Fit stability test

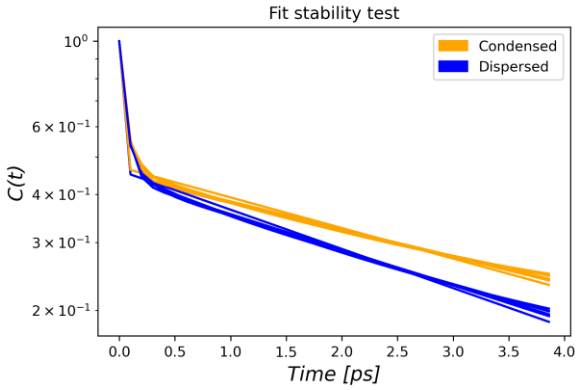

	Condensed	Dispersed
⟨τ [ps]⟩	3.05±0.17	2.25±0.07

Supplementary Figure 10. Fit stability for the analysis of the correlation functions from atomistic molecular dynamics. A series of 250 random parameters were selected as initial guesses for the triple exponential fit, the fits that demonstrated unphysical behavior were removed from the analysis, then the weighted average constant of the function was calculated for all the remaining fits. The average of the resulting values for the condensed and dispersed phase is denoted as ⟨τ [ps]⟩.

Supplementary References

(1) Roeters, S. J.; Strunge, K.; Pedersen, K.; Golbek, T.; Bregnhøj, M.; Wang, Y.; Dong, M.; Otzen, D.; Nielsen, J.; Schiøtt, B.; Weidner, T. Direct Evidence for Upright and Helical Structure of Alpha-Synuclein at Lipid Membranes. *Nat. Commun.* **2023**, *14*, 5731.

(2) Roeters, S. J.; Van Dijk, C. N.; Torres-Knoop, A.; Backus, E. H. G.; Campen, R. K.; Bonn, M.; Woutersen, S. Determining in Situ Protein Conformation and Orientation from the Amide-I Sum-Frequency Generation Spectrum: Theory and Experiment. *J. Phys. Chem. A* **2013**, *117*, 6311–6322.
